# Long-term administration of a commercial supplement enriched with bioactive compounds does not affect feed intake, health status, and growth performances in beef cattle

**DOI:** 10.5194/aab-65-135-2022

**Published:** 2022-04-01

**Authors:** Marica Simoni, Arianna Goi, Erika Pellattiero, Alexandros Mavrommatis, Eleni Tsiplakou, Federico Righi, Massimo De Marchi, Carmen L. Manuelian

**Affiliations:** 1 Department of Veterinary Science, University of Parma, Via del Taglio 10, 43126, Parma, Italy; 2 Department of Agronomy, Food, Natural resources, Animals and Environment, University of Padova, Viale dell'Università 16, 35020, Legnaro, Italy; 3 Laboratory of Nutritional Physiology and Feeding, Department of Animal Science, School of Animal Biosciences, Agricultural University of Athens, Iera Odos 75, 11855 Athens, Greece

## Abstract

Feed additives including natural bioactive compounds (BCs) in combination with vitamin E (VitE) and organic Se could mitigate animal stress associated with intensive livestock farming due to their anti-inflammatory and antioxidant properties. Yeast and yeast derivate are included in feed additives as probiotic products and digestion promoters. *Scutellaria baicalensis* is a source of bioactive compounds and has been tested in monogastrics, exhibiting many immunostimulating and hepato-protective activities. However, the literature lacks information regarding *S. baicalensis* effects on beef cattle performance and health status. The aim of the present study was to evaluate the impact on beef cattle's feed intake, health and oxidative status, and growth performances of the inclusion of a commercial supplement (CS) containing VitE, organic Se, yeast derivate, and *S. baicalensis* extract during the fattening and finishing period. A total of 143 Charolaise male cattle were allotted into 12 pens of 11–12 animals each and assigned to a control ( 463.9±21.48 body weight – BW) or a treated ( 469.8±17.91 BW) group. Each group included two replicates of three pens. The treated groups were supplemented with 20 $g\;\mathrm{CS}\;{\mathrm{animal}}^{-1}\;d^{-1}$ . Feed intake was measured monthly on a pen base during two consecutive days. Total mixed ration and fecal samples were collected at three time points (monthly, from November to February) and pooled by replicate for the analyses to monitor digestibility. Blood samples were individually collected at the beginning and at the end of the trial for oxidative status and metabolic profile determination. Final BW and carcass weight were individually recorded to calculate average daily gain, feed conversion ratio, and carcass yield. Similar feed digestibility between groups were observed during the whole experiment. Feed intake, growth performances, final body weight, average daily gain, feed conversion rate, oxidative status, and metabolic profile were not affected by the dietary inclusion of the tested CS indicating no detrimental effect of the treatment. Different doses of this product should be tested in the future in order to provide a more complete report on the product efficacy.

## 1 Introduction

Intensification of livestock farming can be associated with several management, environmental, and nutritional stressors that increase animals' susceptibility to disease (Bhimte et al., 2021; Endris et al., 2021; Fernandez-Novo et al., 2020). However, antibiotics use in livestock diets as growth promoters or to reduce the rumen dysfunctions is restricted, which has led to the recruitment and study of new natural feed additives to improve growth and feed efficiency (Balcells et al., 2012; Dang et al., 2021). Hence, there is an interest in testing natural bioactive compounds capable of expressing anti-inflammatory and antioxidant activities to enhance livestock health and performance as well as to improve product quality (Righi et al., 2021, Tsiplakou et al., 2021, Pitino et al., 2021, Manuelian et al., 2021). Bioactive compounds can be associated with minerals and vitamins that potentially enhance their action. Micronutrients such as Se, and especially its organic form, help to keep the correct functionality of the immune system participating in the antioxidant defenses (Sgoifo Rossi et al., 2020) and are involved in the thyroid hormone metabolism affecting growth performances and health status in a dose-dependent mode (Mehdi and Dufrasne, 2016). Dietary vitamin E (VitE), a well-known antioxidant, modulates immune system function (Lewis et al., 2019). Similar effects have been summarized for dry yeast, cell wall yeast, and other yeast derivatives (Broadway et al., 2015; Shurson, 2018), which are added as supplements in animal feed due to their high nutritional value and their content in nutraceutical bioactive compounds (e.g. β -glucans, mannanoligosaccharides, nucleotides) that have been demonstrated to improve animal health, immune status, and growth performance (Shurson, 2018).

Recently, Chinese medicinal herbs have attracted the attention of many researchers as possible sources of natural bioactive compounds in animals' production. The roots of *Scutellaria baicalensis* have traditionally been used in Chinese herbal medicine to treat liver and lung diseases, hypertension, acute respiratory infections, acute gastroenteritis, infantile diarrhea, and morning sickness (Zhao et al., 2019; Nurul et al., 2011). The bioactive compounds isolated from this plant are flavonoids, terpenoids volatile, oils, and polysaccharides, which exhibit many activities and effects on the immune system and liver protection (Zhao et al., 2019). The four main flavonoids of *S. baicalensis*, namely baicalein, baicalin, wogonoside, and wogonin, have demonstrated anti-tumor, antibacterial, antiviral, antioxidant, antihypertensive, and hepato-protective effects when individually evaluated (T. Huang et al., 2019; Huynh et al., 2020; Olagaray et al., 2019; Vergun et al., 2019), which suggests a potential beneficial effect of the whole plant on the animal organism.

In fact, the antioxidants and anti-inflammatory properties of *S. baicalensis* have been tested in pigs (Dang et al., 2021), and in chickens (at 0.1 %, 0.5 %, and 1.5 % of the diet) and broilers (at 0.1 %, 0.5 %, and 1.5 % of the diet; Króliczewska et al., 2004, 2008, 2017; Kwon et al., 2009). The dietary inclusion of a highly bioavailable form of *S. baicalensis* extract obtained by enzymatic bio-conversion by pure enzyme or whole cell culture showed comparable effects with apramycin antibiotic on growth performance, nutrient digestibility, fecal microbiota, fecal gas emission, and blood hematology and improved pigs' average daily gain (ADG) and final body weight (BW) (Dang et al., 2021). Additionally, it has been demonstrated that the dietary supplementation with *S. baicalensis* in piglets decreased diarrhea frequency and increased the feed conversion ratio (C. Huang et al., 2019). The inclusion of *S. baicalensis* extract or root also increased the final BW and feedstuff consumption in broilers (Króliczewska et al., 2004, 2008; Kwon et al., 2009) without affecting meat quality and chemical composition (Króliczewska et al., 2008). On the other hand, an excess of *S. baicalensis* root impaired the development of the immune organs in chickens (Króliczewska et al., 2017).

In ruminants, some evidence indicates that *S. baicalensis* could interact with the rumen ecology, altering rumen microbiome, i.e. increasing the prevalence of the phylum Firmicutes, Clostridia class, and *Ruminococcaceae* family (Yausheva et al., 2019), which suggests a possible effect on diet digestibility. Recent literature reports that flavonoids, as powerful antimicrobials, can increase ruminal volatile fatty acids production, while reducing ammonia and methane production (Kalantar, 2018); however, in vitro results were not always consistent (Balcells et al., 2012). The dietary long-term supplementation (60 d) with *S. baicalensis* extract reduced somatic cell counts and increased milk yield in early lactating cows (Olagaray et al., 2019). However, few studies have been conducted on ruminants (Olagaray et al., 2019). Thus, *S. baicalensis* bioavailability and its effect on production, health, and metabolic profile need to be further explored through in vivo trials, particularly in beef cattle, where, to the best of our knowledge, only one study tested *S. baicalensis* (Song et al., 2014). This study demonstrated the positive effect of a mix of herbs including *S. baicalensis* on digestibility and growth performance in beef cattle when evaluated under heat-stress conditions.

The aim of the present study was to evaluate the effect on beef cattle intake, growth performances, and metabolic profile of the addition of a commercial supplement (CS) including VitE, organic Se, and *S. baicalensis* during the fattening and finishing periods.

## 2 Materials and methods

### 2.1 Product tested

The ingredients of the CS for young cattle tested in the present study (IAR4045 AXION BEEF SE; ITACA Europa SA, Italy), as declared by the manufacturer, were calcium carbonate, yeast products, and *S. baicalensis* extract and included 75 000 $\mathrm{UI}\;{\mathrm{kg}}^{-1}$ of VitE, 20 $\mathrm{mg}\;{\mathrm{kg}}^{-1}$ of hydroxylated analog of selenomethionine, and aromatic substances. The mineral content of the CS was determined through spectrophotometric analysis using an ICP-AES spectrophotometer model ULTIMA 2 (HORIBA Jobin Yvon Inc., 3880 Park Avenue, Edison, NJ, USA) after microwave mineralization performed through the Milestone system, model MLS-1200 MEGA (Gemini sustainable laboratory equipment, 7312 DG Apeldoorn, the Netherlands). Chemical composition analysis of the CS was performed as described below in the “Sampling and measurements” section. Mineral content and chemical composition are reported in Table 1. The manufacturer-recommended dose to be administered per animal was 20 $g\;d^{-1}$ .

**Table 1:** Gross composition (mean) and detailed macromineral and micromineral ( mean±standard deviation) characterization of the tested commercial supplement.

Trait	Content
Gross composition	
DM ${}^{*}$ , % as fed	94.67
Crude protein, % DM	2.77
Ether extract, % DM	0.13
Crude fiber, % DM	1.27
Ash, % DM	86.75
Macromineral content	$g\;{\mathrm{kg}}^{-1}$
Ca	189.00±3.00
Mg	4.55±0.06
S	5.61±0.05
P	0.69±0.01
Na	5.21±0.02
K	2.82±0.01
Micromineral content	$\mathrm{mg}\;{\mathrm{kg}}^{-1}$
Zn	420±10
Cu	19.3±0.6
Fe	9420±80
Mn	194±1
Se	17±2
Si	170±10

${}^{*}$ DM: dry matter.

### 2.2 Experimental design, animals, and housing

The study was carried out in a feedlot located in northern Italy (latitude 45.6705, longitude 12.4212). A total of 143 male Charolaise cattle imported from France were involved in the study and monitored throughout the fattening and finishing period for 159.7 d (SD, 3.5 d) from October 2019 to the first half of March 2020. The animals were allotted to 12 pens, each one containing 11 or 12 animals, and were previously fed with an adaptation diet for the first month (October 2019). Then, a fattening diet was administered for the next 2 months and a finishing diet for the rest of the trial. The diets, whose compositions are reported in Table 2, were formulated to meet the nutritional requirements suggested in the NRC (2016) for beef cattle. At the beginning of the fattening period the pens were assigned to one of two dietary groups: control (CON; age: 307.86±22.87 d; BW: 465.15±21.42 kg) and treatment (CS; age: 304.86±22.89 d; BW: 467.48±19.56 kg), which received 20 $g\;d^{-1}$ per animal of the CS product as a top dressing in the feedbunk according to the manufacturer recommendations. Each thesis was replicated in two subgroups of three pens. Animals of both groups were slaughtered at the end of the trial on 6 and 13 March due to the schedule of the slaughterhouse.

**Table 2:** Ingredients (kg per animal as feed) and chemical composition ( mean±standard deviation) of fattening and finishing beef cattle total mixed rations administered during the trial.

Trait	Fattening diet	Finishing diet
Ingredient composition	Kilogram per animal as feed	
Feedstuff ${}^{1}$	1.4	1.4
Distiller	1.0	0.9
Corn gluten meal	0.5	0.4
Alfalfa hay, 15 % of CP	0.3	0.3
Corn meal	3.0	
Cane molasses	0.7	0.8
Wheat straw	0.4	0.4
Earlage ${}^{2}$	1.8	1.8
Dry beet pulp	0.6	0.5
Corn silage ${}^{3}$	7.0	6.8
Triticum silage ${}^{4}$	1.0	1.0
Chemical composition ${}^{5}$	Mean±standard deviation	
Dry matter (DM), % of feed	59.99±1.64	59.62±1.41
CP, % DM	12.90±0.39	12.93±0.30
aNDFom, % DM	30.38±2.15	29.94±1.00
ADFom, % DM	15.28±1.31	13.01±0.73
Lignin (sa), % DM	2.75±0.64	3.53±1.21
Starch, % DM	32.96±2.11	36.51±2.30
Ash, % DM	6.41±0.72	6.34±0.61
Ca, % DM	0.811±0.07	0.943±0.05
P, % DM	0.289±0.00	0.298±0.01
Mg, % DM	0.174±0.00	0.174±0.01
Cl, % DM	0.575±0.10	0.427±0.03
K, % DM	0.836±0.01	0.825±0.02
Na, % DM	0.236±0.04	0.218±0.03
S, % DM	0.218±0.01	0.195±0.02
Fe, % DM	206.4±35.21	232.2±22.01
Cu, % DM	9.85±0.17	11.3±0.99
Zn, % DM	78.23±7.58	80.8±6.10
Mn, % DM	40.85±0.75	49.1±4.23
Si, % DM	1.32±0.06	1.45±0.06
DCAD ${}^{6}$ , $\mathrm{mEq}\;{\mathrm{kg}}^{-1}$	1.85±0.72	6.43±2.70

${}^{1}$ Feedstuff provided in a pelleted form and composed by rapeseed meal, soybean meal 46 % CP, sunflower meal, corn gluten meal, wheat middlings, wheat meal, calcium carbonate, sodium chloride, cane molasses, dry extract of *Aspergillus oryzae*, *Yucca schidigera*, hydrolyzed lignocellulose, sodium bicarbonate, corn meal, magnesium oxide, yeasts. ${}^{2}$ Earlage, 64 % DM, 58 % starch, 14 % NDF. ${}^{3}$ Corn silage, 34 % DM, 33 % starch, 44 % NDF. ${}^{4}$ Triticum silage, 30 % DM, 50 % NDF, 29 % NFC. ${}^{5}$ Analyzed chemical composition – aNDFom: neutral detergent fiber assayed with a heat-stable amylase and expressed exclusive of residual ash; ADFom: acid detergent fiber expressed exclusive of residual ash; lignin(sa): lignin determined by solubilization of cellulose with sulfuric acid; CP: crude protein. ${}^{6}$ DCAD: dietary cation–anion difference.

### 2.3 Sampling and measurements

Feed intake was calculated on a pen basis once a month from November to February for two consecutive days by measuring the difference between the amount of feed delivered and residues. A sub-sample (1 kg) of the total mixed ration (TMR) offered to the animals was collected monthly from November to February from each pen during the feed delivery and pooled by a subgroup of three pens (replicate) for their chemical analysis. Fecal samples were also collected monthly from November to January from each pen and pooled by replicate for their posterior analysis. The fecal samples were collected from the rectum from at least five animals per pen to get a representative sample from each pen.

Chemical composition of the TMR and fecal samples were determined as described in Simoni et al. (2021). Briefly, samples were dried at 55 ${}^{\circ }C$ for 48 h (for TMR) and 72 h (for feces) and then ground in a Cyclotec mill (Tecator, Herndon, VA, USA) to pass a 1 mm screen. The dry matter (DM) content was determined by drying the samples at 103 ${}^{\circ }C$ overnight, and the fiber fractions were analyzed and expressed as aNDFom (neutral detergent fiber assayed with a heat-stable amylase and expressed exclusive of residual ash), ADFom (acid detergent fiber expressed exclusive of residual ash) and lignin(sa) (lignin determined by solubilization of cellulose with sulfuric acid). All the fiber fractions were assayed in duplicate. The nitrogen (N) content was measured by the combustion digestion of the samples at 900 ${}^{\circ }C$ in excess of oxygen by Dumatherm® (Gerhardt GmbH & Co, Königswinter, Germany). Ash content was determined by ignition at 550 ${}^{\circ }C$ . The starch content in the TMR samples was determined by the polarimetric method. The undigested NDF (uNDF) was obtained by fermentation of 240 h in an in vitro batch system using rumen fluid collected at the slaughterhouse from five adult cows. The uNDF was used for the estimation of the in vivo apparent total-tract digestibility of nutrients as described by Righi et al. (2017) and Simoni et al. (2021). The mineral content of the TMR was determined by X-ray fluorescence according to Berzaghi et al. (2018).

Individual beef BW was measured at the beginning and at the end of the trial to calculate the average daily gain (ADG). The feed conversion ratio (FCR) was calculated at the beginning of the fattening period and at the end of the trial as a ratio between the DM intake and the BW. The health status of the animals was assessed regularly by visual evaluation, and pathologies detected were classified as “lameness”, “respiratory disease”, and “other health issues”. The specific antibiotic administered to each animal was also recorded (length of the treatment and drug used).

Individual blood samples were simultaneously collected during the official sanitary check at the beginning of the trial and during slaughter using 10 mL vacuum tubes containing lithium heparin (Terumo Venosafe 10 mL VF-109SHLTerumo Europe L.V., Leuven, Belgium). Plasma was extracted from blood samples by centrifugation at 3000 g for 15 min. The plasmatic α -tocopherol concentration was determined by an iCheck fluorometer–spectrophotometer (iCheck Vitamin E; BioAnalyt GmbH, Teltow, Germany). The metabolic profile of plasma samples included bilirubin, aspartate aminotransferase (AST-GOT), gamma glutamyltransferase (GGT), creatine kinase, total protein, albumin, urea, glucose, total cholesterol, triglycerides, and minerals (Ca, Mg, and P). Non-esterified fatty acids (NEFA) and β -hydroxybutyrate acid (BHBA) were also determined to monitor the energy balance of the animals. The total antioxidant capacity (FRAP: ferric reducing ability power; ABTS: 2,2'-azino-bis (3-ethylbenzothiazoline-6-sulfonic acid)) and catalase (CAT) activity were determined spectrophotometrically (GENESYS 180, Thermo Fisher Scientific, Massachusetts, USA) in plasma, aiming to assess the oxidative status of the animals (Tsiplakou et al., 2018).

At the end of the trial, all cattle were transported to a commercial slaughterhouse. The BW at slaughter and the carcass weight were recorded individually, and the proportion of carcass yield was calculated as the proportion of the carcass weight on the BW at slaughter. Due to the Covid-19 outbreak, it was not possible to perform measurements and sampling on organs and carcass except for the weight of the latter that was provided by the electronic scale of the slaughterhouse.

### 2.4 Statistical analyses

Sources of variation of performance traits were investigated using the GLM procedure of SAS version 9.4 (SAS Inst. Inc., Cary, NC) according to the following linear model:

1 $$y_{ijkl}=\mu +{\text{feeding}}_{i}+{\text{box}}_{j}({\text{feeding)}}_{i}+i{\text{BW}}_{k}+\varepsilon_{ijkl},$$

where $y_{ijkl}$ is the dependent variable; μ is the overall intercept of the model; ${\text{feeding}}_{i}$ is the fixed effect of the i th CS supplementation ( i=1 and 2, where 1 means non-supplemented and 2 means supplemented); ${\text{box}}_{j}(\text{feeding}{)}_{i}$ is the fixed effect of the j th box ( j=1 to 12) nested within the i th CS supplementation; iBWk is the fixed effect of the k th initial BW class ( k=1 to 3, where 1=iBW≤460kg ; 2=460kg>iBW≤475kg ; 3=iBW>475kg ); and $\varepsilon_{ijkl}$ is the random residual $∼N(0,\sigma_{e}^{2})$ , where $\sigma_{e}^{2}$ is the residual variance. The feeding effect was tested using “box within feeding” as the error term, whereas the other fixed effects were tested on the residual.

For the comparison of the slaughter weights, the same model was used but the initial body weight was introduced as covariate in the model.

Sources of variation of the TMR and feces chemical composition as well as diet digestibility were investigated using the GLM procedure of SAS according to the following linear model:

2 $$y_{ijkl}=\mu +{\text{feeding}}_{i}+{\text{box}}_{j}({\text{feeding)}}_{i}+{\text{Time}}_{k}+\varepsilon_{ijkl},$$

where $y_{ijkl}$ is the dependent variable; μ is the overall intercept of the model; ${\text{feeding}}_{i}$ is the fixed effect of the i th CS supplementation ( i=1 and 2, where 1 means non-supplemented and 2 means supplemented); ${\text{box}}_{j}(\text{feeding}{)}_{i}$ is the fixed effect of the j th box ( j=1 to 4) nested within the i th CS supplementation; ${\text{Time}}_{k}$ is the fixed effect of the k th time class ( k=1 to 4, where 1=November , 2=December ; 3=January , and 4=February ); and $\varepsilon_{ijkl}$ is the random residual $∼N(0,\sigma_{e}^{2})$ , where $\sigma_{e}^{2}$ is the residual variance. The feeding effect was tested using “box within feeding” as the error term, whereas the other fixed effects were tested on the residual.

Sources of variation of the blood parameters were investigated using the GLM procedure of SAS according to the following linear model:

3 $$y_{ijkl}=\mu +{\text{feeding}}_{i}+{\text{box}}_{j}({\text{feeding)}}_{i}+\varepsilon_{ijkl},$$

where $y_{ijkl}$ is the dependent variable; μ is the overall intercept of the model; ${\text{feeding}}_{i}$ is the fixed effect of the i th CS supplementation ( i=1 and 2, where 1=non supplemented; 2=supplemented ); ${\text{box}}_{j}(\text{feeding}{)}_{i}$ is the fixed effect of the j th box ( j=1 to 4) nested within the i th CS supplementation; and $\varepsilon_{ijkl}$ is the random residual $∼N(0,\sigma_{e}^{2})$ , where $\sigma_{e}^{2}$ is the residual variance. The feeding effect was tested using box within feeding as the error term, whereas the other fixed effects were tested on the residual.

Sources of variation of FRAP, ABTS, and CAT were investigated using the GLM procedure of SAS using the initial value as covariate and the dietary treatment was the fixed effect. For all the models, multiple comparison of least-square means was performed for the fixed effects using Bonferroni's test ( P<0.05 ).

The frequency of health issues was compared between groups using the chi square test; the health issues were expressed as the sum of lameness, respiratory problems, and other health problems recorded. Due to the low number of animals that needed a treatment, these results are reported as total number of cases.

## 3 Results

Few animals in both groups presented health issues during the whole study (17 out of 143 beef cattle) that needed antibiotic treatment. Because the overall health status of these animals was acceptable and abnormal behavior was not observed, we decided to keep the treated animals within the study. The statistical analysis performed on the sum of health issues did not show significant differences between groups.

A physiological increase in feed intake (on a DM basis) was observed moving from the fattening to the finishing period ( P<0.001 ). On average, daily overall intake was around 10 kg of DM per animal, moving from 9.88±0.104 to 10.45±0.104 kg of DM per beef cattle in the fattening and finishing period, respectively ( P<0.001 ). No significant differences were observed between feeding groups (Table 3).

**Table 3:** Average individual feed intake and growth performance traits (least-square mean and standard error of the mean – SEM) of control (CON) and commercial supplement (CS) groups.

Trait	CON	CS	SEM	P value
Intake, kg DM	10.22	10.14	0.131	0.658
Intake in the fattening period, kg DM	9.87	9.89	0.147	0.269
Intake in the finishing period, kg DM	10.58	10.39	0.147	0.269
Body weight at slaughter, kg	722.34	720.03	3.652	0.712
Carcass weight, kg	441.10	437.98	2.380	0.577
Carcass yield, % ${}^{1}$	61.10	60.83	0.173	0.637
ADG ${}^{2}$	1.60	1.58	0.019	0.709
FCR ${}^{3}$	10.27	10.29	0.077	1.000

${}^{1}$ Calculated as percentage of live body weight at slaughter. ${}^{2}$ ADG: average daily gain. ${}^{3}$ FCR: feed conversion ratio.

A similar fecal composition was observed between the two dietary groups ( P>0.05 ). The average fecal DM was 17.94 % ( P=0.451 ), while the average content of CP ( P=0.928 ), aNDFom ( P=0.412 ), ADFom ( P=0.468 ), and lignin(sa) ( P=0.143 ) was 17.2 %, 51.2 %, 25.7 %, and 6.74 % of DM, respectively. Moreover, a similar apparent total-tract digestibility of DM and NDF (% on DM) between groups was obtained (DM, 70.8 % and 73.59 % for CON and CS groups, respectively, P=0.411 ; NDF: 50.42 % and 54.98 % for CON and CS groups, respectively, P=0.498 ).

**Table 4:** Plasmatic oxidative status, and metabolic profile (least-square mean and standard error of the mean – SEM) of animals in the control (CON) and commercial supplement (CS) groups.

Trait ${}^{*}$	CON	CS	SEM	P value
Oxidative status				
α -Tocopherol, $\mathrm{mg}\;L^{-1}$	3.11	3.54	0.101	0.202
FRAP, µM ascorbic acid	0.65	0.72	0.030	0.528
ABTS, inhibition %	29.7	29.5	0.229	0.768
CAT, $\mathrm{units}\;{\mathrm{mL}}^{-1}$	2.76	2.55	0.099	0.303
Metabolic profile				
Total protein, $g\;L^{-1}$	74.36	73.81	0.551	0.646
Albumin, $g\;L^{-1}$	33.55	33.96	0.270	0.622
Globulin, $g\;L^{-1}$	40.81	39.84	0.518	0.427
Albumin / globulin ratio	0.83	0.86	0.014	0.356
Urea, $\mathrm{mmol}\;L^{-1}$	2.90	2.81	0.087	0.543
NEFA, $\mathrm{mmol}\;L^{-1}$	0.31	0.23	0.013	0.158
Glucose, $\mathrm{mmol}\;L^{-1}$	6.48	6.41	0.162	0.635
Total cholesterol, $\mathrm{mmol}\;L^{-1}$	2.74	2.75	0.073	0.532
Triglycerides, $\mathrm{mmol}\;L^{-1}$	0.16	0.18	0.005	0.690
AST-GOT, $\mathrm{IU}\;L^{-1}$	78.86	80.64	1.158	0.938
GGT, $\mathrm{IU}\;L^{-1}$	24.17	25.15	0.571	0.395
Bilirubin, $µ\mathrm{mol}\;L^{-1}$	1.18	0.78	0.064	0.223
Creatine kinase, $\mathrm{IU}\;L^{-1}$	365.43	439.73	27.270	0.307
${\mathrm{Ca}}^{2+}$ , $\mathrm{mmol}\;L^{-1}$	2.57	2.62	0.027	0.196
$P^{3+}$ , $\mathrm{mmol}\;L^{-1}$	2.65	2.63	0.035	0.730
${\mathrm{Mg}}^{2+}$ , $\mathrm{mmol}\;L^{-1}$	1.02	1.01	0.019	0.607
BHBA, $\mathrm{mmol}\;L^{-1}$	0.27	0.27	0.007	0.861

${}^{*}$ FRAP: ferric reducing ability of plasma; ABTS: 2,2'-azino-di(3-ethylbenzthiazoline-6-sulfonic acid); CAT: catalase; NEFA: nonesterified fatty acids; AST-GOT: aspartate aminotransferase; GGT: gamma glutamyltransferase; BHBA: β -hydroxybutyric acid.

Performance traits retrieved at slaughter showed that dietary supplementation with CS did not affect the BW at slaughter (Table 3), which was on average 721 kg. The tested product did not affect carcass weight, carcass yield, ADG, or FCR (Table 3), being on average 439.54 kg, 60.96 %, 1.59 $\mathrm{kg}\;d^{-1}$ , and 10.28 $\mathrm{kg}\;(\mathrm{kg}\;\mathrm{BW}{)}^{-1}$ , respectively. The dietary supplementation with CS did not impair blood α -tocopherol content, antioxidant status, CAT, or the metabolic profile (Table 4). On average, blood α -tocopherol content, total antioxidant capacity (with FRAP and ABTS assays), and CAT activity were respectively 3.30 $\mathrm{mg}\;L^{-1}$ , 0.66 µM ascorbic acid, 29.78 % inhibition, and 2.56 units ${\mathrm{mL}}^{-1}$ , whereas NEFA, total cholesterol, and triglycerides content were 0.28, 2.65, and 0.17 $\mathrm{mmol}\;L^{-1}$ , respectively. Similar results between groups were also observed for the liver parameters CK and BHBA, which average values where 388.38 $\mathrm{IU}\;L^{-1}$ and 0.26 mmol L ${}^{-1}$ , respectively.

## 4 Discussion

Despite the increasing interest of livestock producers and feed additive suppliers in using natural feed additives to improve livestock health and performance as a consequence of the ban in the European Union of growth promoters in 2006 (European Commission, 2003), in vivo trials information on their use is still lacking, in particular when referring to non-poultry species. Few studies have been conducted with *S. baicalensis*, the majority having been on pigs (Dang et al., 2021; C. Huang et al., 2019) and chickens (Króliczewska et al., 2004, 2008, 2017; Kwon et al., 2009) and only one on beef (Song et al., 2014). Therefore, the present study contributes to increasing the scientific knowledge on the inclusion of bioactive compounds from *S. baicalensis* during the fattening and finishing phases when rearing beef cattle. It should be mentioned that data collection on the organs and carcass traits, including meat quality and composition, in order to completely evaluate the effects of the addition of CS was not possible due to the Covid-19 outbreak and the subsequent legal restrictions to movement of people and to access to the slaughterhouse.

The chemical composition of the fattening and finishing diets in the present study were in line with those reported by other authors in Charolaise beef cattle (Cortese et al., 2019, Sgoifo Rossi et al., 2019). The NDF and CP levels were particularly similar to those reported by Cortese et al. (2019), and starch level variation was comparable to those indicated by Sgoifo Rossi et al. (2019). The addition of the CS did not significantly modify the chemical composition of the diet. This aspect is important to make comparable results from both groups, CON and CS. Moreover, feed intake observed in the fattening and finishing phases is consistent with the physiological growth of the animals, and it is comparable to the findings from both Cortese et al. (2019) and Sgoifo Rossi et al. (2019) on similar animals. Furthermore, the level of intake observed in our study, combined with the diet energy and protein amount, was the expected one to allow the relatively high ADG observed. These results support that the addition of the product as a top dressing in the feedbunk did not impair the TMR palatability (Baumont, 1996). Our results are in agreement with the ones reported by Swecker et al. (2008) showing that parenteral administration of two doses of Se or/and VitE did not affect ADG in weaned beef calves. On the other hand, Sgoifo Rossi et al. (2020) reported that the mineral supplementation with organic Se along with other organic minerals (Zn, Cu, and Mn) improved BW and ADG in Charolaise beef during the fattening period but achieved a similar FCR of those supplemented with minerals from an inorganic source.

Although *S. baicalensis* has been suggested to modify rumen microbiome increasing Firmicutes phylum, Clostridia class, and the *Ruminococcaceae* family in an in vivo experiment (Yausheva et al., 2019), in our study we did not see an effect on diet digestibility which might be related to the low doses of bioactive compounds, minerals, and vitamins in the tested product. In fact, there is an absence of differences in the estimated total tract apparent digestibility of nutrients of the animals. Moreover, the absence of differences in digestibility is in contrast with conclusions of Shurson (2018) in his review on yeast and yeast derivatives' effects on ruminant digestion. In fact, the latter author reported that in many studies performed on ruminants the supplementation with yeast or yeast products improved fiber intake and digestion, enhancing cellulolytic and fibrolytic growth.

The supplementation with yeast and their derivatives demonstrated to improve growth performances in several studies conducted on ruminants (Broadway et al., 2015; Shurson, 2018). On the other hand, pigs supplemented with *S. baicalensis* extract improved ADG, final BW (Dang et al., 2021), and FCR (C. Huang et al., 2019) and reduced diarrhea frequency (C. Huang et al., 2019). In broilers, greater final BW has been reported. However, this improvement can be related to the higher intake observed in the studies (Króliczewska et al., 2004, 2008; Kwon et al., 2009). It is worth mentioning that an excess of *S. baicalensis* root has been reported to reduce the development of the immune organs in chickens (Króliczewska et al., 2017). The only study conducted in beef revealed the positive effect of a mix of herbs including *S. baicalensis* on digestibility and growth performances in beef cattle's body temperature, endocrinological status, blood biochemical parameters, nutrient apparent digestibility, and growth performance under heat-stress conditions (Song et al., 2014). In the present study, we did not find any of these effects when supplementing beef fattening and finishing diets (Table 3) with CS, probably because intake (Table 3), feed chemical composition, and feed digestibility between groups were similar. No effect of the organic Se was detected. This is in agreement with the literature, which explains the lack of visible effects of Se supplementation in several studies on calves, cows, and bulls (Mehdi and Dufrasne, 2016).

In our study, the metabolic profile results were within the normal range for beef animals (Alcantara et al., 2020) and similar between dietary groups. Also, plasmatic levels of VitE, which were within the normal range for beef cattle (Hidiroglou et al., 1988), did not differ between groups even if the CS also included VitE. This agrees with Swecker et al. (2008), who did not find VitE plasmatic differences either, despite administering the two doses of VitE or/and Se to calves via parenteral, whereas O'Grady et al. (2001) found an increase in plasma and in muscle α -tocopherol content in cattle fed 300 IU of α -tocopherol per kilogram of feed, with a decreased lipid and oxymyoglobin oxidation. The latter author reported no differences in plasma Se content in cattle fed 0.3 $\mathrm{mg}\;{\mathrm{kg}}^{-1}$ of organic Se and also no differences in the susceptibility to lipid and oxymyoglobin oxidation in presence or absence of VitE. The amount of Se supplemented in the present study was probably too low to see an effect; moreover, since blood Se is bound to globulins ( α - and β -), albumin, and low- and very low-density lipoproteins (Mehdi and Dufrasne, 2016) that were not affected by CS supplementation, the Se transport itself would not be enhanced in this case. The β -glucans in the yeast cell wall may improve immune competence in young animals; they can adsorb or bind toxins, viruses, and several pathogenic bacteria, while other components of the yeast cell wall (e.g. mannanoligosaccharides) serve as prebiotics and have antioxidant and anti-mutagenic effects (Shurson, 2018). Similarly, Se yeast supplements enhanced antioxidant status, immune responses, and anti-radicalic activity (Mehdi and Dufrasne, 2016; Sgoifo Rossi et al., 2017), while VitE as an exogenous antioxidant improves cellular membrane stability. Several flavonoids have been demonstrated to have the capacity to trap free radicals, including reactive nitrogen species and reactive oxygen species (ROS) as well as chelating metals, whereas phenolic acids act as antioxidants mainly by scavenging free radicals (Manuelian et al., 2021). Furthermore, baicalin included in *S. baicalensis* at high doses induced a reduction in ROS production associated with increased intracellular concentrations of catalase in bovine mammary epithelial cells (Perruchot et al., 2019). Despite the promising effects of this bioactive compounds, in the present study there were no differences between the two groups, neither in the total antioxidant capacity nor in catalase activity of blood plasma.

## 5 Conclusions

The results of the present study revealed that the supplementation with a CS that includes VitE, organic Se, yeast derivative, and *S. baicalensis* added as a top dressing during the fattening and finishing period did not impair beef cattle feed intake and digestibility. Moreover, supplementation did not have a detrimental or beneficial impact on growth performances. Plasmatic VitE levels of FRAP, ABTS, and CAT activity were not modified due to the supplementation with CS, and the animals' metabolic profile remained similar. Different doses of this CS, as well as its potential effects on other blood parameters and on animal products, should be tested to provide a more complete report on the product efficacy.

## Data Availability

The data presented in this study are available free of charge for any user upon reasonable request from the corresponding author.
